# When to vaccinate a fluctuating wildlife population: Is timing everything?

**DOI:** 10.1111/1365-2664.13539

**Published:** 2019-12-31

**Authors:** Courtney L. Schreiner, Scott L. Nuismer, Andrew J. Basinski

**Affiliations:** ^1^ Department of Mathematics University of Idaho Moscow ID USA; ^2^ Department of Biological Sciences University of Idaho Moscow ID USA

**Keywords:** fluctuating population, Lassa, oral vaccination, seasonal reproduction, timing of vaccination, tuberculosis, wildlife vaccination, zoonotic disease

## Abstract

1. Wildlife vaccination is an important tool for managing the burden of infectious disease in human populations, domesticated livestock and various iconic wildlife. Although substantial progress has been made in the field of vaccine designs for wildlife, there is a gap in our understanding of how to time wildlife vaccination, relative to host demography, to best protect a population.
2. We use a mathematical model and computer simulations to assess the outcomes of vaccination campaigns that deploy vaccines once per annual population cycle.
3. Optimal timing of vaccination is an important consideration in animals with short to intermediate life spans and a short birthing season. Vaccines that are deployed shortly after the birthing season best protect the host population.
4. The importance of timing is greater in wildlife pathogens that have a high rate of transmission and a short recovery period. Vaccinating at the end of the birthing season best reduces the mean abundance of pathogen‐infected hosts. Delaying vaccination until later in the year can facilitate pathogen elimination.
5. *Policy Implications*. Tuning wildlife vaccination campaigns to host demography and pathogen traits can substantially increase the effectiveness of a campaign. Our results suggest that, for a fluctuating population, vaccinating at, or shortly after, the end of the birthing season, best protects the population against an invading pathogen. If the pathogen is already endemic, delaying vaccination until after the birthing season is over can help facilitate pathogen elimination. Our results highlight the need to better understand and predict host demography in wildlife populations that are targeted for vaccination.

## Graphical abstract

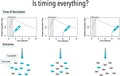

## 1 INTRODUCTION

Controlling infectious diseases that circulate in wildlife populations remains an important goal. Sixty per cent of emerging diseases in humans (e.g. Ebola, Lassa) originated as zoonoses that transmitted primarily in wildlife (Jones et al., 2008). In addition to the risks posed to humans, zoonoses like tuberculosis and brucellosis impose substantial financial burdens on farming industries by infecting livestock (Chambers et al., 2014; Phillips & Van Tassell, 1996). Finally, zoonoses like brucellosis can harm iconic animals like bison and elk in Yellowstone National Park (Davis et al., 1990; Rhyan et al., 1994; Williams, Thorne, Anderson, & Herriges, 1993).

Vaccination has proven itself an effective tool for reducing the burden of infectious disease in diverse wildlife populations (Freuling et al., 2013; MacInnes et al., 2001; Sidwa et al., 2005). Vaccines distributed in bait form have been instrumental in eliminating and/or mitigating strains of rabies in Europe (e.g. Arctic fox [Freuling et al., 2013]) and North America (e.g. gray fox, coyotes [Sidwa et al., 2005], red fox [MacInnes et al., 2001], raccoons [Maki et al., 2017]). Furthermore, vaccination can provide a means of disease control in iconic species like bison and elk for which other methods of disease control (i.e. culling) are not appropriate (Davis & Elzer, 2002; Olsen, 2013). Currently, oral vaccine technology is being developed for several diseases in a variety of wildlife (e.g. brucellosis in various wildlife [Olsen, 2013; Davis & Elzer, 2002], tuberculosis in badgers [Corner et al., 2010], and plague in prairie dogs [Rocke et al., 2010]).

A central challenge for wildlife vaccination is the development of cost‐effective vaccination strategies that best protect a population against an invading pathogen. The effectiveness of a vaccination campaign is measured by the fraction of the population that the campaign immunizes (Maki et al., 2017). One important consideration for achieving a high population immunity in wildlife is the timing of vaccine delivery relative to seasonal fluctuations in host abundance (Boyer, Canac‐Marquis, Guérin, Mainguy, & Pelletier, 2011; Masson, Bruyére‐Masson, Vuillaume, Lemoyne, & Aubert, 1999; Vos et al., 2001). In Europe, for example, vaccination campaigns that target fox populations in the fall generally immunize a greater proportion of the host population, when compared to campaigns that distribute vaccine in the spring or summer (Masson et al., 1999; Vos et al., 2001). Similarly, vaccination campaigns that target raccoons in the fall experience a greater rate of vaccine uptake than campaigns in the spring or summer (Boyer et al., 2011). In both cases, vaccination campaigns that distribute vaccine earlier than fall risk missing the current year's juvenile population, primarily because the juveniles are not yet foraging for food. Intuitively, a similar vaccine delivery strategy will be necessary in other wildlife reservoirs that undergo seasonal reproduction. However, the relative importance of timing for other zoonotic reservoirs is unknown.

Although less well‐recognized, the dynamics of wildlife populations may also create novel opportunities for pathogen control. Theoretical work suggests that large population fluctuations, such as those that occur in animals with a short life span and rapid seasonal reproduction (e.g. rodents), lessen the fraction of the population that must be vaccinated to achieve eradication (Peel et al., 2014). Alternatively, targeting fluctuating populations at their seasonal low points could maximize the impact of small numbers of vaccine baits. Finally, a campaign that distributes vaccine when the population does not have an influx of susceptible hosts may have a greater proportional influence on the pathogen's ability to spread. Although many such benefits could, in principle, be realized, identifying the optimal timing of vaccine delivery involves a complex interplay of numerous factors.

In this work, we explore the importance of timing a vaccination campaign relative to host population dynamics as well as properties of the targeted pathogen. Specifically, we simulate the outcome of a vaccination campaign that targets a wildlife population that fluctuates in size due to seasonal reproduction. We begin by assessing the general importance of timing in vaccine delivery when the goal of the campaign is to prevent a pathogen from invading the population. Next, we simulate scenarios in which the pathogen is endemic in the population, and ask how different pathogen traits influence the importance of timing in various wildlife. To more clearly interpret our results, we evaluate vaccination scenarios with parameters chosen to simulate two specific host‐pathogen systems. The first, multimammate rats *Mastomys natalensis*, serve as a primary reservoir of Lassa fever in humans (Lecompte et al., 2006). Populations of *M. natalensis* exhibit extreme annual fluctuations in population size in some regions (Leirs et al., 1997) that could influence the importance of timing vaccination campaigns. The second reservoir we focus on is the European badger *Meles meles*, which acts as a relatively long‐lived reservoir of tuberculosis in livestock populations (Cheeseman, Wilesmith, & Stuart, 1989). These host–pathogen systems allow a comparison of the importance of timing for zoonotic diseases with a short (Lassa virus) and a long (tuberculosis) duration of infection.

## 2 MATERIALS AND METHODS

We model a vaccination campaign applied to a seasonally fluctuating wildlife population. The model consists of a system of ordinary differential equations (ODEs) that partition the host population into non‐overlapping classes. The classes, in turn, track the infection and immunity status of the population with respect to a zoonotic pathogen.

### 2.1 Model

We assume the host population undergoes seasonal reproduction that, in turn, results in a well‐defined birthing season. During the birthing season, susceptible newborns are introduced at a constant rate *b*_0_, independent of the current population size. The function *b*(*t*) describes the birthing rate during the annual birthing season, which in the model begins on the first day of every year, and lasts *t*_b_ days. To describe this mathematically, we use the modulus function, notated mod (*t*, 365), that expresses time *t* as time (days) into the current year. With this notation, the birth function *b*(*t*) can be written

(1) $$b\left(t\right)=\left\{\begin{array}{cc} b_{0} & \;\mathrm{mod}\;\;\left(t,\;365\right)\leq t_{b}\\ 0 & \;\mathrm{mod}\;\;\left(t,\;365\right)>t_{b}. \end{array}\right.$$

In contrast to the seasonal nature of reproduction, mortality is assumed to be constant across the year, with all hosts dying at a constant per capita rate *d*. This combination of seasonal reproduction and constant mortality leads to stable population cycles characterized by an annual increase in population size followed by an annual decrease in total population size. We assume that all newborn hosts are susceptible to the target pathogen (class *S*). If infected with the pathogen, susceptible hosts transition into the pathogen‐infected class (*I*_p_).

Let *N* denote the total population size. The per capita rate of susceptible infection is specified through the force of infection, notated $\lambda (I_{p},\;N)$. We explore both frequency‐dependent ($\lambda \left(I_{p},\;N\right)=\beta_{p}\frac{I_{p}}{N}$) and density‐dependent ($\lambda \left(I_{p},\;N\right)=\beta_{p}I_{p}$) modes of transmission (Keeling & Rohani, 2011). These modes describe two extreme views of how transmission scales with the density of pathogen‐infected hosts: in density‐dependent transmission, the force of infection scales linearly as the density of infected hosts increases; in frequency‐dependent transmission, the force of infection increases with the fraction of infected hosts. We also incorporate the possibility of pathogen virulence. At rate $\gamma_{p}$, infected hosts transition into a recovered class (*P*) with probability $1-p_{\mu }$, and die from infection with probability $p_{\mu }$. In the model, hosts that have recovered from the pathogen maintain lifelong immunity to subsequent pathogen infection.

Our model applies an annual, pulse‐style vaccination campaign. On day *t*_v_ of each year, the campaign exposes *n*_v_ hosts in the population to the vaccine. In line with existing vaccination programmes that use vaccine baits, we assume that vaccine exposure is distributed randomly among seronegative and seropositive hosts. As a consequence, some vaccines are used on hosts that have previously developed immunity to the pathogen, either because of prior pathogen infection or prior vaccination. Letting *S* and *N* denote the densities of susceptible and total hosts at time *t*, vaccination is described by

(2) $$\sigma \left(t,\;S,\;N\right)=\delta \left(\mathrm{mod}\;\left(t,\;365\right)-t_{v}\right)\min\left(n_{v}\frac{S(t)}{N(t)},\;S\left(t\right)\right).$$

The Dirac‐*δ* notation implies that, at times *t*_v_ into each year, the value of the state variable *S* is instantaneously decreased by the value returned by the min() function. In turn, the min() function constrains the number of vaccinations to be less than the number of susceptible individuals currently present in the population.

Upon contact with the vaccine, susceptible hosts transition to an intermediate immune state (*S*_v_). Class *S*_v_ describes hosts that have begun to mount an immune response, but have not yet acquired immunity to the pathogen. Hosts that have been exposed to the vaccine acquire lifelong pathogen immunity at rate $\gamma_{v}$, and transition into class *V*. These biological assumptions lead to the following system of ODEs:

(3a) $$\frac{dS}{dt}=b\left(t\right)-\lambda \left(I_{p},\;N\right)S-dS-\sigma \left(t\right)$$

(3b) $$\frac{dS_{v}}{dt}=\sigma \left(t\right)-\lambda \left(I_{p},\;N\right)S_{v}-\left(\gamma_{v}+d\right)S_{v}$$

(3c) $$\frac{dI_{p}}{dt}=\lambda \left(I_{p},\;N\right)S+\lambda \left(I_{p},\;N\right)S_{v}-\left(d+\gamma_{p}\right)I_{p}$$

(3d) $$\frac{dV}{dt}=\gamma_{v}S_{v}-dV$$

(3e) $$\frac{dP}{dt}=\left(1-p_{\mu }\right)\gamma_{p}I_{p}-dP.$$

Although Equation (2) formally describes the vaccination process, in our numerical simulations, vaccination is implemented as a recurring jump discontinuity in the state variables of System (3). Specifically, whenever $\;\mathrm{mod}\;\left(t,\;365\right)==t_{v}$, simulation of System (3) stops, and the state variables *S* and *S*_v_ are updated according to

(4) $$\begin{array}{c} S\left(t\right)=S\left(t\right)-\min\left(n_{v}\frac{S(t)}{N(t)},\;S\left(t\right)\right)\\ S_{v}\left(t\right)=S_{v}\left(t\right)+\min\left(n_{v}\frac{S(t)}{N(t)},\;S\left(t\right)\right), \end{array}$$

whereafter simulation of System 3 continues. This procedure is implemented in the statistical language R, using the ‘desolve’ package (Soetaert, Petzoldt, & Setzer, 2010). Parameters and state variables are summarized in Table 1.

We use this model to evaluate the effectiveness of different timings in two scenarios. In the first, the pathogen is assumed absent from the population. In this case, the state variables *I*_p_ and *P* are equal to zero, and the corresponding equations, (3c) and (3e), are omitted from our simulations. Here, we focus on timing strategies that minimize the inefficiencies of long‐term vaccine bait programmes in different wildlife species. In the second scenario, the pathogen is endemic in the host population. Here, we investigate the effect that timing of vaccination has on both the mean number of pathogen‐infected hosts as well as the probability of eliminating the pathogen from the population.

**Table 1:** State variables, parameters and functions in the model. See text for details on the parameter values

Name	Description
*S*	Susceptible hosts (state variable)
*S*_v_	Vaccine‐exposed hosts (state variable)
*I*_p_	Pathogen‐infected hosts (state variable)
*V*	Vaccinated hosts (state variable)
*P*	Pathogen‐recovered hosts (state variable)
*b*_0_	Birth rate during birthing season
*t*_b_	Duration of birthing season
*d*	Natural mortality rate
$\lambda (I_{p},\;N)$	Function specifying force of infection
$\gamma_{v}$	Rate of immunity after vaccine exposure
*n*_v_	Number of vaccines
$\beta_{p}$	Pathogen transmission
$\gamma_{p}$	Rate of transition out of pathogen‐infected class
$p_{\mu }$	Probability of virulence‐induced mortality

### 2.2 Strategies that prevent a pathogen's invasion

To gauge the extent to which different timings of vaccination ward off an invading pathogen, we use the reproduction number of the pathogen, *R*_0_ *_,_*_p_, to develop a measure of the pathogen's ability to invade a regularly vaccinated population. Here, *R*_0_ *_,_*_p_ is defined as the number of secondary infections that occur over the course of an annual population cycle, when an infected host is introduced into a completely susceptible population (Keeling & Rohani, 2011). When annual vaccination occurs, a related quantity, termed the *realized* reproduction number, $R_{0,p}^{*}$, gives a similar time‐averaged measure of how vulnerable the population is to pathogen invasion.

We evaluate the fractional reduction in the average rate at which a pathogen invades the vaccinated population, relative to the case in which vaccination does not occur. If a frequency‐dependent force of infection is assumed, the fractional reduction in the pathogen's time‐averaged ability to invade an annually vaccinated population is

(5) $$f=1-\frac{R_{0,p}^{*}}{R_{0,p}}=\frac{1}{T}\int_{0}^{T}\frac{V^{*}\left(t\right)}{N^{*}\left(t\right)}dt$$

(Appendix S1). Appendix S1 contains details on the analogous form of the reduction under density‐dependent transmission. The superscript * denotes state variables that have reached a stable limit cycle, so that the time‐dependent solutions V*(*t*) and N*(*t*) are periodic with period equal to 1 year. Note that because Equation (5) describes the reduction in annual pathogen transmission relative to the annual rate of transmission without vaccination, we characterize the extent to which vaccination reduces the pathogen's ability to invade a population without the need to specify pathogen transmission or recovery.

We use Equation (5) to define an optimal window of vaccination for various wildlife hosts. Defining the optimal timing of vaccination as that which best reduces the pathogen's rate of invasion, the optimal window is the time period for which at least 95% of the optimal reduction is realized.

### 2.3 Controlling an endemic pathogen

Here, we investigate how timing of vaccination influences the ability of a campaign to control a pathogen that is already circulating in a wildlife population. To understand the optimal vaccine delivery strategy in this scenario, we first calculate the mean annual abundance of infected hosts in the absence of any vaccination, and after the host classes have settled into stable cycles. Next, we calculate the mean abundance of infected hosts when annual vaccination campaigns are implemented. We report the fractional reduction in the mean number of infected hosts, given the type of transmission, pathogen parameters and timing of the annual vaccination campaign.

In addition to exploring how general pathogen traits influence the importance of timing in vaccination, we also present simulations specific to two zoonotic reservoirs: multimammate rats *Mastomys natalensis*, which act as the primary reservoir for Lassa virus, and badgers *Meles meles*, an important reservoir of tuberculosis. To better understand the optimal timing for pathogen elimination in these specific reservoirs, we use simulations of the ODE model outlined above as well as stochastic simulations that describe System (3) as a Poisson process using the Gillespie algorithm (Gillespie, 1977). The latter simulations are analogous to System (3) with different events (e.g. birth, death, vaccination, pathogen infection) occurring at different probabilistic rates according to the terms in the ODE system (Table 2).

**Table 2:** Transitions in the stochastic model. With the exception of vaccination, only one event can occur per time step. During a pulse vaccination, multiple hosts transition from *S* to *S*_v_. *N* denotes total population size

Event	Probabilistic rate
Host birth	See Equation (1)
Natural host death	*dN*
Death from pathogen infection	$p_{\mu }\gamma_{p}I_{p}$
$S\to S_{v}$	See Equation (4)
$S\to I_{p}$	*λS*
$S_{v}\to I_{p}$	*λS*_v_
$I_{p}\to P$	$\gamma_{p}\left(1-p_{\mu }\right)I_{p}$
$S_{v}\to V$	$\gamma_{v}S_{v}$

We use stochastic simulations to investigate how campaign timing influences the probability of eliminating a pathogen, and the degree to which the abundance of pathogen‐infected hosts can be reduced. For each simulation, we initialize the state variables to the values that are predicted by the deterministic version of the model, when vaccination is absent and the pathogen is allowed to circulate to quasi steady state. Once initialized at quasi steady state, the Gillespie algorithm is used to simulate 2 years forward in time without vaccination. This period of time is used to calculate ${\bar{I}}_{0,p}$, the average number of pathogen‐infected hosts in the absence of vaccination. At this point, any simulations in which the pathogen has undergone stochastic extinction are discarded. Simulations are only included in the analysis if the pathogen is still present in the population when vaccination is started in the third year. Vaccination occurs over the next 5 years. For each year following the first vaccination, we calculate the mean number of infected hosts, termed ${\bar{I}}_{p}$. Across values of *t*_v_ varying from 0 to 365, we calculate the fractional reduction in the abundance of infected hosts as

(6) $$f_{\text{path}}=1-{\bar{I}}_{p}/{\bar{I}}_{0,p}.$$

Similarly, for each year following the onset of vaccination, we track the number of simulations in which the pathogen was eliminated. The fractional reduction and probability of elimination for years one, three and five are calculated. To better understand the stochastic nature of these results, we run 500 simulations of each parameter set for *Mastomys natalensis* and 50 simulations for *Meles meles*. We use more simulations in the *Mastomys natalensis* system because the outcome of vaccination was more variable. Using those simulations in which the pathogen persisted until vaccination was started, we then use bootstrapping to calculate a 95% confidence interval of simulation outcome.

### 2.4 Parameterization

We use parameters that broadly describe vaccination campaigns that target multimammate rats within a village area and badgers in an agricultural setting. We investigate scenarios in which the number of vaccine exposures is equal to one half and one fourth the average size of the targeted population.

#### 2.4.1 Multimammate rat

Each rodent has a 1‐year life span. Although shorter life spans are often estimated from capture–mark–recapture studies (100–200 days), seasonal rodent movement between sites likely biases these estimates to be lower than true life span (Fichet‐Calvet, Becker‐Ziaja, Koivogui, & Günther, 2014; Mariën, Kourouma, Magassouba, Leirs, & Fichet‐Calvet, 2018; Mariën, Sluydts, et al., 2018). We choose the birth rate to reflect an average village population of 2,000 rodents (Mariën et al., 2019). Seasonal reproduction begins in June and lasts about 4 months (Holt, Davis, & Leirs, 2006; Leirs et al., 1997). We choose *t*_b_ = 120 to model the resulting 4‐month birth period.

Although the epidemiological details of Lassa virus in *Mastomys* are still being discovered, empirical studies have shown that infection is relatively nonvirulent, and that in the closely related Morogoro virus, the typical duration of viral shedding is 18–39 days (Borremans et al., 2015). We set $\gamma_{p}=\frac{1}{30}$ to describe a mean recovery time of 30 days. Virulence is set to zero ($p_{\mu }=0$). The proportion of rodents exposed to Lassa in endemic areas is around 50% (Fichet‐Calvet et al., 2014). In a non‐fluctuating population, classical results from epidemiology show that the fraction of the population that is affected by the pathogen is $1-\frac{1}{R_{0,p}}$ (Keeling & Rohani, 2011). We use this information to estimate that $R_{0,p}=2$, which allows us to uniquely determine the transmission coefficient $\beta_{p}$.

#### 2.4.2 Badger

Life span is set to 4 years, typical of those reported in Gloucestershire county of South West England (Wilkinson et al., 2000). We simulate a badger population in a 50‐km^2^ region. At typical densities of 20 km^−2^, this implies an average population size of 1,000 individuals in the targeted area (Cheeseman et al., 1989). Recruitment of cubs begins in February and typically occurs over a 2‐month period (Cheeseman et al., 1989; Nowak & Walker, 1999). To this end, we choose *t*_b_ = 60 days. Badgers that are infected with tuberculosis typically exhibit long periods of latent infection with low virulence, which are often followed by an active period of infection with high virulence (Wilkinson et al., 2000). Here, we parameterize our model with high virulence $p_{\mu }=1$. Because the effect of virulence is low at the population level, we assume that death from tuberculosis occurs at a rate equal to the natural mortality rate (Cheeseman et al., 1989). Consequently, the average life span of an infected badger is 2 years. We set $R_{0,p}=1.5$, resulting in an endemic infection that broadly matches empirical measures of prevalence (30%–40%; Cheeseman et al., 1989).

## 3 RESULTS

### 3.1 Strategies that prevent a pathogen's invasion

**Figure 1:**
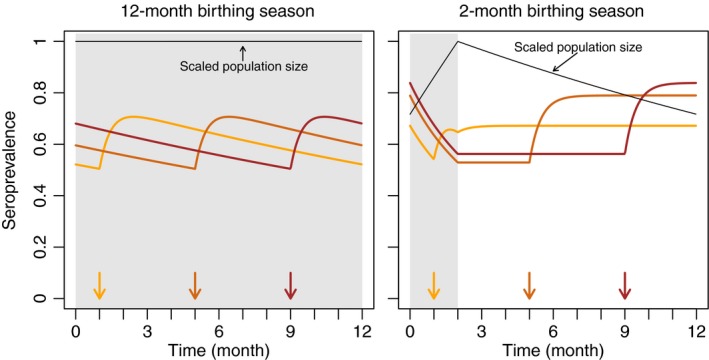
Annual seroprevalence profile in two regularly vaccinated populations. Arrows denote the times of vaccination and the grey region indicates the hosts' birthing season. Time is scaled relative to the start of the birthing season. Host life span is set to 2.5 years. The birth rate is set so that the peak population size is 1,000. Each pulse vaccination exposes 500 hosts to the vaccine. Susceptible hosts that are exposed to the vaccine develop immunity after a 2‐week period ($\gamma_{v}=0.07$)

A primary goal of wildlife vaccination is to preempt the establishment of a pathogen by regularly vaccinating an uninfected population (Maki et al., 2017). Figure 1 shows how vaccine‐induced annual seroprevalence changes throughout the year in two host populations that differ in the length of their birthing season. For a fixed time of vaccination, the host seroprevalence that is predicted by our model exhibits stable, periodic cycles that we use to analyse the importance of timing. In a population that breeds year‐round, our model predicts that seroprevalence continually decreases as newborns are added to the population and increases following each pulse vaccination. Changing the timing of vaccination shifts the seroprevalence profile, but does not change its underlying shape (Figure 1). The seroprevalence profile of a host with a well‐defined (i.e. short) birthing season shows two key differences. First, seroprevalence is constant at times when neither birthing nor vaccination is taking place. In our model, this occurs because no newborns are being added to the population at these times and mortality targets seronegative and seropositive hosts equally. Second, in a fluctuating population, shifting the time of vaccination changes the minimum and maximum seroprevalence realized during an annual cycle (Figure 1). This occurs because the application of a fixed number of vaccines has a greater effect when applied during those times of the year when population size is small.

**Figure 2:**
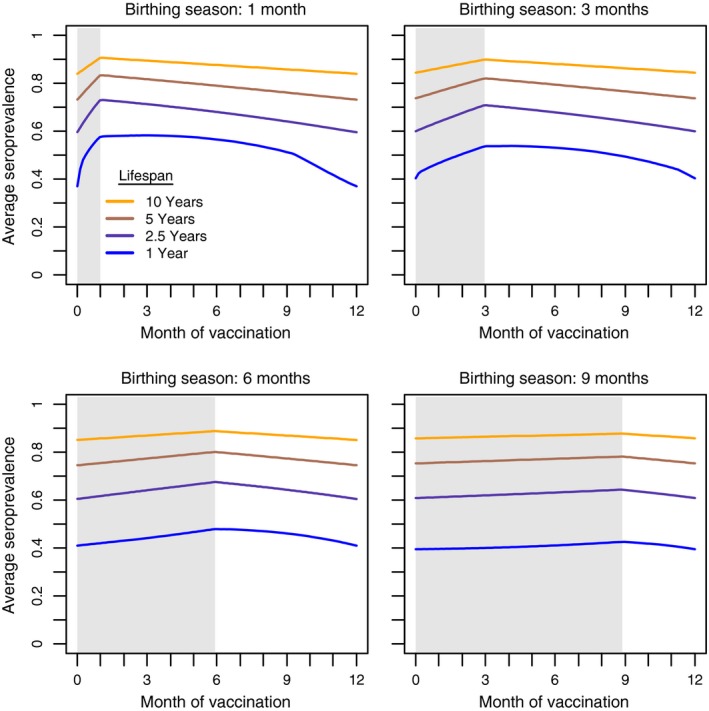
Yearly averaged seroprevalence in a regularly vaccinated population with varying host demography. Month of vaccination is scaled relative to the start of the birthing season (grey region). In all scenarios, the number of vaccines is set to 500 and the peak population size is 1,000. Other parameters: $\gamma_{v}=0.07$

Both the host's life span and the duration of the birthing season influence the outcome of vaccinating at different times of the seasonal cycle (Figure 2). Our results show that, across life spans that range from 1 to 10 years, the optimal time to vaccinate is immediately after the birthing season. Using epidemiological theory outlined in Appendix S1, we show that, if transmission is frequency‐dependent, the yearly averaged seroprevalence is a measure of the fractional reduction in the pathogen's ability to spread in the population throughout the year. By this definition of protection, our results imply that, in a stably cycling host population, a pulse vaccination that occurs immediately at the end of the birthing season causes the greatest reduction in the pathogen's ability to spread (Figure 2). When pathogen transmission is density‐dependent, the fractional reduction on the pathogen's *R*_0_ *_,_*_p_ is given by the mean number of vaccinated hosts divided by the mean population size (Appendix S1). Similarly, when transmission is frequency‐dependent, the optimal time to vaccinate is immediately after the birthing season (Figure S1). At this time, a host population is composed primarily of new susceptible hosts, increasing the likelihood that vaccine baits are distributed to individuals without immunity from previous campaigns.

The importance of achieving the optimal strategy can be ascertained by the slopes of the lines near the optimal vaccination time. Regardless of life span, the greatest potential cost of vaccinating at the wrong time occurs in campaigns that target hosts with a short birthing season (≤3 months) and distribute vaccines before the birthing season is over (Figure 2). Delaying vaccination beyond the end of the birthing season also decreases the level to which the population is protected, but to a lesser extent. For campaigns that err by distributing vaccines shortly after the end of the birthing season, the cost to average seroprevalence is greatest in hosts with an intermediate life span (2.5 years). Hosts with a long life span and/or a long birthing season do not exhibit pronounced population fluctuations from year to year. As a result, the overall influence of timing in a vaccination campaign is less important in long‐lived animals and the cost of vaccinating after the optimal time is minimal (Figure 2). In short‐lived hosts (life span of 1 year), the optimal time to vaccinate is also at the end of the birthing season. However, a short life span lessens the extent to which population immunity can accumulate from previous vaccinations. As a result, there is little cost of vaccinating at a suboptimal time, so long as vaccination occurs at least 3 months before the next birthing season. Animals with an intermediate life span, however, survive long enough to increase population immunity and have high rates of population turnover that change seroprevalence.

**Figure 3:**
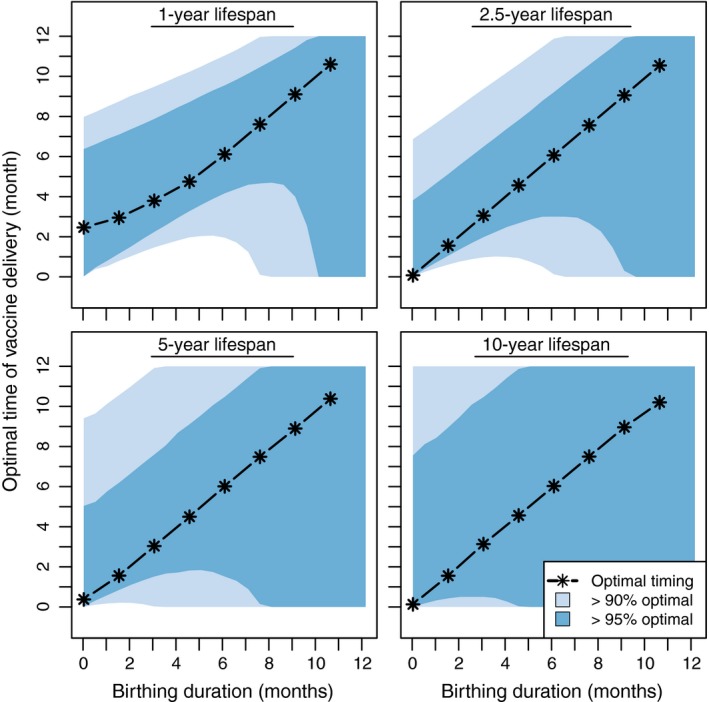
Optimal window of vaccination for hosts with different life spans and birthing season durations. For these scenarios, the number of vaccinations is 500 and the peak population size is 1,000 hosts. Other parameters: $\gamma_{v}=0.07$

Figure 3 shows how the optimal vaccination window changes with the duration of birthing season and life span. For hosts with a 1‐year life span and a birthing season lasting <6 months, the window of vaccination is approximately 5 months. Hosts with a 2.5‐year life span, in contrast, have a window of 6 months when births occur over a 6‐month interval and a window of 3 months when the birthing season occurs over a single month. For longer‐lived organisms, the optimal time of vaccination is less constrained: with a rapid birthing season of just 1 month, a host with a 5‐year life span has a 4‐month optimal window, while a host with a 10‐year life span has 7‐month optimal window. Table 3 displays the optimal window of vaccination for a variety of wildlife hosts for which vaccines are being used, or considered for use (Cross, Buddle, & Aldwell, 2007; Murphy, Redwood, & Jarvis, 2016).

**Table 3:** A non‐exhaustive list of wildlife hosts for which vaccination campaigns are implemented, or being considered (Cross et al., 2007; Murphy et al., 2016). For each host, we list a representative zoonotic disease that motivates the development of a vaccine. We use simulations of the host population with the pathogen absent to calculate the spread around the optimal time of vaccination that achieves 95% of the maximal reduction in a pathogen's ability to invade. The row corresponding to fruit bats was made using *Hypsignathus monstrosus*, *Epomops franqueti* and *Myonycteris torquata*, three candidate reservoirs of Ebola (Leroy et al., 2005). Other parameters: $\gamma_{v}=0.07$

Species	Pathogen	Birthing duration (months)	Life span (years)	Duration of 95% optimal vaccination window (months)	Demographic reference
Multimammate rat *Mastomys natalensis*	Lassa virus	4	1	5.4	See text
Raccoon *Procyon lotor*	Rabies	1.5–5.5	2.5	3.9–5.4	Feldhamer, Thompson, and Chapman (2003)
Red fox *Vulpes vulpes*	Rabies	2–3	2.5	3.9–4.4	Nowak and Walker (1999)
Brush‐tailed possum *Trichosurus vulpecula*	Tuberculosis	2–4	6.5	6.4–7.8	Gilmore (1969); Nowak and Walker (1999)
Eurasian badger *Meles meles*	Tuberculosis	2	4	4.9	Nowak and Walker (1999); Wilkinson et al. (2000)
White‐tailed deer *Odocoileus virginianus*	Tuberculosis	0.5–5	5	4.7–7.3	Feldhamer et al. (2003)
Elk *Cervus canadensis*	Brucellosis	2	10	8.6	Berger and Cain (1999); Evans, Mech, White, and Sargeant(2006)
Feral pig *Sus scrofa*	Pseudorabies	5–12	10	12	Feldhamer et al. (2003); Nowak and Walker (1999)
Big‐horn sheep *Ovis Canadensis*	Pasteurellosis	2–3	6.5	6.4–7.1	Nowak and Walker (1999)
African buffalo *Syncerus caffer*	Tuberculosis	4–12	18	10.8–12	Nowak and Walker (1999)
Bison *Bison bison*	Brucellosis	1–4	10	7.8–10.8	Feldhamer et al. (2003)
Fruit bats (various species)	Ebola	2	10	8.6	Brunet‐Rossinni and Austad (2004); Nowak and Walker (1999)

### 3.2 Controlling an endemic pathogen

**Figure 4:**
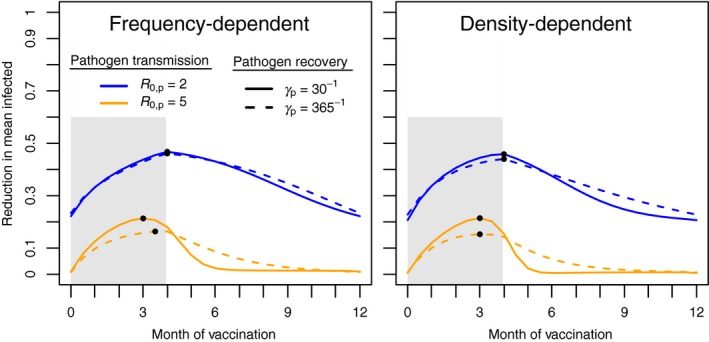
Fractional reduction in the mean number of pathogen‐infected hosts versus timing of vaccination. Left and right graphs show the effect of frequency‐ and density‐dependent transmission. Dots indicate the location of the maximal reduction in infected hosts. Life span is set to 1 year, peak population size is 1,000 individuals and 250 vaccines are used. Other parameters: $p_{\mu }=0$, $\gamma_{v}=0.07$

Up to this point, our simulations have focused on preventing the invasion of a pathogen. Here we extend these results to a scenario where the target pathogen is already endemic in the host population. Figure 4 shows the reduction in the mean number of infected hosts in the population for different times of vaccination, relative to when the population is not vaccinated. Our results demonstrate that both the *R*_0_ *_,_*_p_ and the rate of pathogen recovery influence the optimal time of vaccination (Figure 4). In this parameter regime, the mode of transmission (frequency‐dependent vs. density‐dependent) has a small effect on the optimal strategy. For pathogens with more moderate rates of transmission ($R_{0,p}=2$), the optimal time of vaccination is at the end of the birthing season. As transmission is increased to $R_{0,p}=5$, however, the optimal time of vaccination shifts 2 weeks to 1 month earlier. By delivering the vaccine earlier, a greater fraction of the newborn susceptible population are vaccinated before the pathogen has cycled through the population (Figure 4). Our results show that this strategy is increasingly important for pathogens with a rapid infection time‐scale (i.e. shorter time of pathogen recovery).

Incorporating virulence into the model does not change the overall strategy for timing a vaccination campaign (Figure S2). However, when transmission is frequency‐dependent, the inclusion of virulence increases the importance of vaccination, even in moderately transmissible pathogens ($R_{0,p}=2$) with fast recovery (30 days). Because more pathogen‐infected hosts die from infection, increasing pathogen virulence increases the proportion of the population that is susceptible and increases the rate of transmission to new susceptible hosts. As a result, the fractional reduction that results from late‐year vaccination becomes small because most of the susceptibles have been removed from the population at this point (Figure S2).

### 3.3 Rodent population

**Figure 5:**
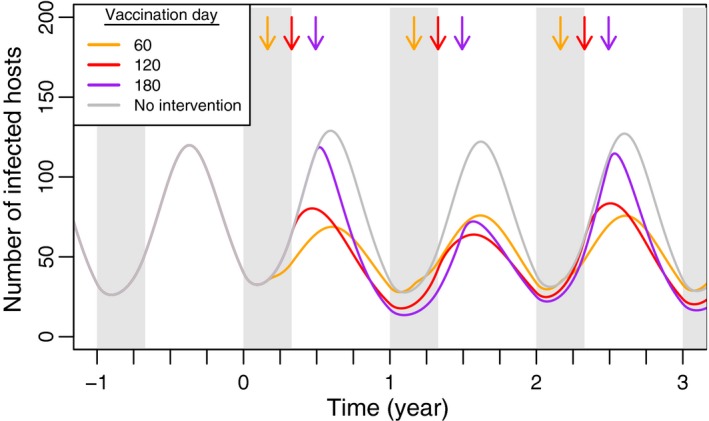
Time course of pathogen‐infected class in rodent simulations. The grey line (‘No Intervention’) indicates the number of infected hosts in the absence of any vaccination. Each coloured line indicates the number of infected hosts when the population is repeatedly vaccinated at times indicated by an arrow of the same colour. Host life span is 1 year and the birthing season is 120 days. The birth rate is set so that the average population size is 2000 individuals, and each pulse vaccination exposes 500 hosts to vaccine. The duration of pathogen infection is 30 days and $R_{0,p}=2$. Other parameters: $\gamma_{v}=0.07$

The multimammate rat *Mastomys natalensis* is the primary reservoir host of Lassa fever (Fichet‐Calvet et al., 2014). Simulations of this system using differential equations demonstrate that timing of vaccination plays an important role in the degree to which an endemic pathogen can be controlled (Figure 5). Our simulations show that distributing vaccines at or before the end of the birthing season curtails the pathogen when its potential for spread is greatest (Figure 5). In contrast, distributing vaccine 2 months after the birthing season ends does not prevent the annual pathogen outbreak in the first year of vaccination. However, our results also suggest that vaccinating at or after the end of the birthing season might facilitate pathogen elimination by removing the susceptible population at a crucial point in the population cycle.

**Figure 6:**
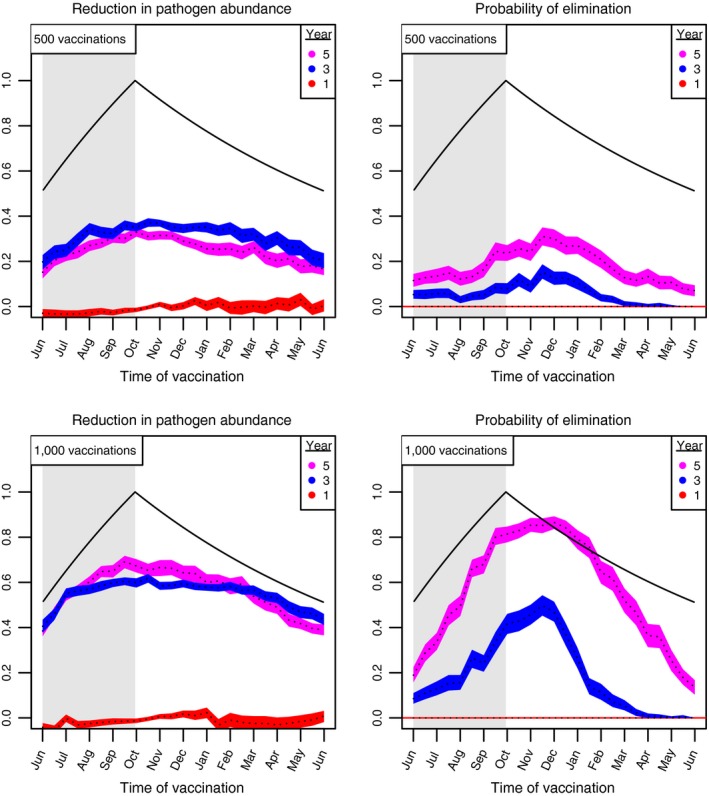
Simulated vaccination outcomes in a rodent population with endemic Lassa virus. The left column shows the reduction in the average number of infected hosts and the right column shows the probability of eliminating the pathogen. Different regions outline a 95% confidence interval of vaccination outcome, with the median represented by a dashed line. Each region corresponds to different years after vaccination is initiated. The pathogen was never eliminated in the first year. The birth rate is set so that the average population size is 2,000 and host life span is 1 year. The duration of pathogen infection is 30 days. Other parameters: $\gamma_{v}=0.07$, $R_{0,p}=2$

When our model is modified to include the possibility of stochastic extinction of the pathogen, our results demonstrate that vaccinating at, or shortly after, the end of the birthing season, is optimal. Figure 6 shows the proportional reduction in pathogen prevalence as well as the probability of pathogen elimination during years one through five of a vaccination programme applied to *Mastomys natalensis* rodents. In campaigns that expose 500 hosts to vaccine, vaccinating at the end of the birthing season facilitates the reduction in the abundance of infected rodents. However, our results also suggest that, with these relatively low levels of vaccine, the probability of eliminating the pathogen is greater when vaccination is delayed up to 2 months beyond the end of the birthing season. If, instead, the campaign has the capacity to expose 1,000 rodents to vaccine during each pulse, a wider range of timings yield substantial reductions in the pathogen's prevalence. Here, vaccinating 2 months prior, or 2 months after, the end of the birthing season, results in substantial reductions in the pathogen's prevalence (Figure 6). In addition to reducing the pathogen's prevalence, vaccinating within 2 months after the end of the birthing season also facilitates pathogen elimination.

Similarly, if pathogen transmission is density‐dependent, our results show that pathogen elimination is most likely to occur when vaccination is applied 1–2 months after the end of the birthing season (Figure S3). In contrast to our simulations that assume frequency‐dependence, the times at which vaccination best reduces the mean number of pathogen‐infected hosts are concentrated at, or before the end, of the birthing season (Figure S3).

### 3.4 Badger population

**Figure 7:**
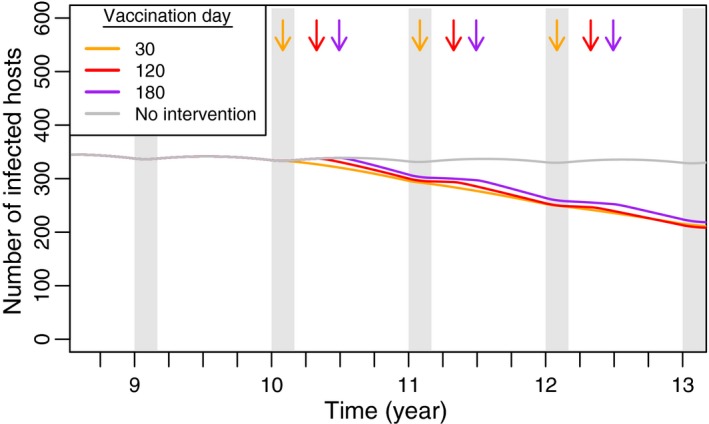
Time course of tuberculosis‐infected class in badger simulations. The grey line (‘No Intervention’) indicates the number of infected hosts in the absence of any vaccination. Each coloured line indicates the number of infected hosts when the population is vaccinated at times indicated by arrow of the same colour. Host life span is 4 years and the birthing season is 60 days. The average population size is set to 1,000 hosts, and each pulse vaccination targets 250 individuals. The average duration of pathogen infection is 2 years, after which hosts die of disease. Other parameters: $\gamma_{v}=0.07$, $R_{0,p}=2$

In long‐lived hosts, the timing of vaccination is less likely to influence the immediate outcome of infectious disease control, especially when pathogen virulence is low. To study this alternative scenario, we set the parameters in our simulations using data from badger populations that serve as a reservoir for tuberculosis. For the parameters we study, simulations predict population dynamics characterized by weak cycles, a prediction consistent with empirical data on badger population dynamics (Cheeseman et al., 1989). Because population sizes do not fluctuate greatly across the year, opportunistically vaccinating at different times throughout the population cycle does not appreciably alter the outcome of the vaccination campaign (Figure 7). With low to moderate levels of vaccine, our results show that vaccinating at different times of the year does not substantially influence the outcome of an annual vaccination campaign, instead only shifting the time course of the pathogen's decline (Figure 7).

**Figure 8:**
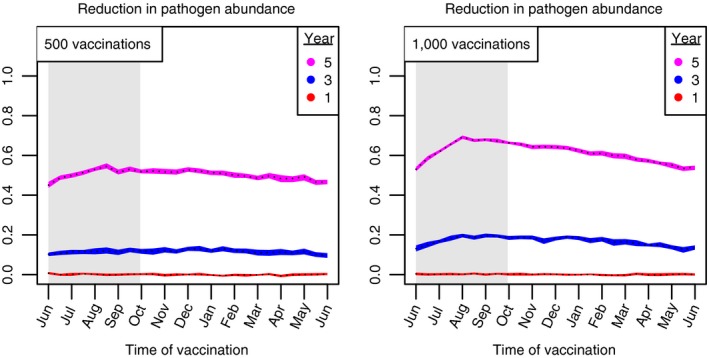
Reduction in the abundance of tuberculosis‐infected badgers across different timings of vaccination. The number of vaccinations is 250 (left pane) and 500 (right pane). Different regions outline a 95% confidence interval of vaccination outcome, with the median represented by a dashed line. Each region corresponds to different years after vaccination is initiated. The birth rate is set so that the average population size is 1,000 hosts and host life span is 4 years. The average duration of infection is 4 years, after which hosts die. Other parameters: $\gamma_{v}=0.07$, $R_{0,p}=1.5$

Our Gillespie simulations verify that changing the timing of vaccination has a lesser effect on the outcome of vaccination, compared to simulations in rodent populations. In these simulations, pathogen elimination never occurred, both because of the longer period of infection that is associated with tuberculosis in badgers and the longer badger life span. However, our simulations imply that, compared to other timings, a campaign that vaccinates at, or before, the end of the birthing season, will better reduce the mean abundance of pathogen‐infected hosts (Figure 8). This trend occurs because vaccination at these times helps preempt the boost in transmission that the pathogen receives from incoming newborns.

## 4 DISCUSSION

The ongoing risk of pathogen spillover from wildlife to humans underscores the need for effective wildlife vaccination strategies. Our results show that the timing of vaccine delivery, relative to a reservoir host's seasonal demographic cycle, can significantly affect the outcome of a vaccination campaign. In campaigns that seek to preempt the invasion of a pathogen, for example, our results imply that the timing of annual vaccination is important for avoiding inefficiencies inherent in campaigns that target hosts at random (i.e. with vaccine baits). Across a broad range of host life spans, the optimal time to vaccinate is immediately at the end of seasonal reproduction when the population size reaches its annual peak. This strategy ensures that vaccines are not wasted on previously vaccinated hosts. Distributing vaccines at this time becomes more important in host populations with short reproductive seasons and short to intermediate (i.e. <2.5 year) life spans.

Our results imply that the timing of vaccine delivery is important when protecting fox and raccoon populations from rabies. These wildlife live long enough that preexisting immunity from prior vaccination campaigns is theoretically possible, and have short breeding seasons that can cause fluctuations in seroprevalence. Our results corroborate empirical findings that fall vaccination is more effective at protecting these host populations than springtime vaccination (Boyer et al., 2011; Masson et al., 1999). This is primarily because juveniles have begun to forage outside of the den at this time and can potentially ingest vaccine baits (Maki et al., 2017). Our results show that a similar optimal vaccination strategy exists in other wildlife as well.

If the pathogen is already endemically cycling in the population, our results indicate that both the traits of the pathogen as well as traits of the host influence the optimal vaccination strategy. Density‐dependent transmission, for example, increases the importance of vaccinating at the optimal time, although the effect is small. The transmission rate, however, has a large impact on the importance of timing, especially when the rate at which hosts clear the pathogen is short. In these cases, vaccinating the population before the end of the birthing period is ideal because it avoids vaccine being consumed by individuals that are already infected with the pathogen.

Our model makes several simplifying assumptions. First and foremost, our model greatly simplifies juvenile development. In fox populations, for example, weaning and/or non‐foraging juveniles do not have access to vaccine baits that are distributed outside of the den site (Boyer et al., 2011; Masson et al., 1999). As a result, a vaccination campaign that distributes baits too early may fail to reach a substantial component of the population (Boyer et al., 2011; Masson et al., 1999). A more complete description of the optimal time to vaccinate will require an explicit description of how vaccine uptake varies with age. Our results suggest that if vaccination cannot take place at the peak population size, then withholding vaccination until later in the year (but before the next breeding season) is optimal.

Maternal antibodies may also lower the efficacy of juvenile vaccination (Zhi & Hildegund, 1992). In this case, the optimal timing of vaccination is shifted beyond the end of the birthing season to a time when vaccines are effective in juveniles (Blasco et al., 2001; Maki et al., 2017). Understanding how the efficacy of a vaccine changes with age will be critical to developing vaccination strategies across diverse wildlife. More generally, our model assumes that host immunity is perfect and lifelong. In reality, a major hurdle confronted by many wildlife vaccination programmes is the development of vaccines that invoke robust and lifelong host immunity (Davis & Elzer, 2002; Olsen, 2013). We anticipate that incorporating waning immunity would decrease the importance of timing in vaccine delivery.

Our model also simplifies the spatial structure of the host population as well as the spatial distribution of vaccines following a campaign. For example, juveniles that disperse may be especially difficult to target after leaving a den site. Similarly, features of the habitat influence when and where adults spend time in their environment and, as a result, likely influence the optimal spatial distribution of vaccine (Rees, Pond, Tinline, & Bélanger, 2013). In raccoon populations, fall vaccination campaigns are effective in part because hosts are foraging aggressively in an effort to acquire fat stores for the upcoming winter (Boyer et al., 2011). Because of this, developing vaccination simulations that incorporate temporal changes in habitat use by the host are essential.

Our model simplifies host population dynamics by assuming that a population is stably cycling due to a single annual breeding season with a constant population birth rate as well as a constant death rate. Because of these simple forms for birth and death, our model likely does not capture the full spectrum of population fluctuations that are possible with density‐dependent growth and death nor can it describe populations with bimodal birth distributions. Better population dynamics models will also help understand the effect of density‐ versus frequency‐dependent transmission, and might also allow more detailed exploration on the effects of pathogen virulence. Furthermore, our simulations with the pathogen endemic assume that the pathogen is stably cycling in the population. Other modelling work has shown that the exact phase of an epidemic affects the outcome of vaccination (Newton et al., 2019). Incorporating more realistic pathogen invasion scenarios will be essential to developing a more fine‐scale timing strategy.

Despite its simplifications, our model clarifies when the timing of vaccination is likely to matter for wildlife vaccination. Generally, in populations that fluctuate annually in size, focusing vaccination efforts to occur immediately after the birthing season helps facilitate pathogen control by targeting the population when population is composed mostly of susceptible hosts. When coupled with other technological advances enhancing the efficacy of wildlife vaccines (e.g. transmissible vaccines), well‐timed vaccine delivery may facilitate the preemptive eradication of human pathogens from their wildlife reservoirs.

## AUTHORS' CONTRIBUTIONS

C.L.S., S.L.N. and A.J.B. conceived the ideas and designed methodology; C.L.S. and A.J.B. coded the simulations and made the figures; C.L.S. and A.J.B. led the writing of the manuscript. All authors contributed critically to the drafts and gave final approval for publication.

## Data Availability

Data available via the Zenodo Digital Repository https://doi.org/10.5281/zenodo.3529849 (Basinski & Schreiner, 2019).
