# Supplementary material for: When to vaccinate a fluctuating wildlife population: Is timing everything?
Source: J Appl Ecol. 2019 Dec 31;57(2):307–19. doi: 10.1111/1365-2664.13539 (PMC7043377; doi:10.1111/1365-2664.13539)
Supplement: Supplementary file 4 [file JPE-57-307-s004.pdf]

Appendix

Derivation of $R_{0,p}$ and $R^*_{0,p}$ assuming frequency-dependent transmission

In this section, we derive expressions for $R_{0,p}$ and $R^*_{0,p}$ assuming a frequency-dependent mode of transmission. Under this assumption, the rate at which hosts experience infectious contacts depends on the fraction of the population that is infected with the pathogen.

In the calculation for $R_{0,p}$, we assume that the population is stably cycling, and composed almost entirely of susceptible hosts. In addition, we assume an infinitesimal density of pathogen-infected hosts, $I_p << 1$, which give rise to an infinitesimal density of pathogen-recovered hosts, $P << 1$, over a single period. In what follows, we assume the susceptible population is near the stable limit cycle solution, and write the variable S as a sum of the limit cycle solution and the deviation from that cycle, $S(t) = S^*(t) + s(t)$. Likewise, the infected population is near 0, motivating the form $I_p(t) = 0 + i_p(t)$ with $i_p(t) << 1$. Next, we integrate the equation for $\frac{dI_p}{dt}$ over a single period to determine whether the number of pathogen-infected individuals grows or decays (Keeling and Rohani, 2011).

In the absence of vaccination, the equation for pathogen-infected hosts is

$$\frac{dI_p}{dt} = \beta_p\, S\, \frac{I_p}{S + I_p + P} - (d + \gamma_p) I_p \tag{1}$$

Note that, because the number of infected individuals is of order $i_p(t)$, the number of pathogen-recovered individuals can be written as a small deviation from 0, $P(t) = 0 + p(t)$. Here, $p(t)$ is of similar magnitude as $i_p(t)$. Plugging in the above forms of S, I_p, and P yields

$$\frac{di_p}{dt} = \beta_p\, (S^* + s)\, \frac{i_p}{S^* + s + i_p + p} - (d + \gamma_p) i_p \tag{2}$$

Keeping only those terms that are first order results in

$$\frac{di_p}{dt} = \beta_p\, i_p - (d + \gamma_p) i_p \tag{3}$$

Dividing both sides of (3) by i_p, and integrating from $t = 0$ to $t = T$ gives a discrete-time map that shows how an initial number of pathogen-infected individuals changes over one period.

$$i_p(T) = i_p(0) \exp\left[\, (\beta_p - (d + \gamma_p))\, T\right] \tag{4}$$

Eq (4) implies that pathogen invasion is determined by

$$R_{0,p} = \frac{\beta_p}{d + \gamma_p}. \tag{5}$$

Specifically, if $R_{0,p} < 1$, the pathogen decays and cannot invade the population, while if $R_{0,p} > 1$, the pathogen invades and remains endemic.

The expression for the realized reproduction number, $R^*_{0,p}$, is derived in a similar manner, but with the addition of vaccinated classes S_v and V in the population cycle. In this case, the equation that describes how an initial pathogen invasion changes over one period is derived in a similar manner, and is equal to

$$i_p(T) = i_p(0) \exp\left[\int_0^T \beta_p \frac{S^* + S^*_v}{N^*} - (d + \gamma_p)\, \mathrm{d}\tau \right], \tag{6}$$

where τ is a dummy variable that integrates over time, and $N^*(t)$ denotes the total population size on the

stable limit cycle. Eq (6) implies that the pathogen will spread when

$$\int_0^T \beta_p \frac{S^* + S_v^*}{N^*} - (d + \gamma_p)\ \mathrm{d}\tau > 0. \tag{7}$$

This allows us to conclude that

$$R_{0,p}^* = \frac{\beta_p}{d + \gamma_p} \frac{1}{T} \int_0^T \frac{S^* + S_v^*}{N^*}\ \mathrm{d}\tau \tag{8}$$

Now, we substitute $N^* - V^* = S^* + S_v^*$ in the expression for $R_{0,p}^*$ to find

$$\begin{aligned} f_{freq} &= 1 - \frac{R_{0,p}^*}{R_{0,p}} & (9) \\ &= 1 - \frac{1}{T} \int_0^T 1 - \frac{V^*}{N^*}\ \mathrm{dt} & (10) \\ &= \frac{1}{T} \int_0^T \frac{V^*}{N^*}\ \mathrm{dt} & (11) \\ &= \overline{(V^*/N^*)}. & (12) \end{aligned}$$

The overhead bar notation in Eq (12) denotes an average over a single population cycle. Thus, the fractional reduction in the pathogen's $R_{0,p}$ is given by the average prevalence of vaccinated hosts at the stable limit cycle. We numerically evaluate the fractional reduction in $R_{0,p}$ by first simulating System 3 of the main text until the state variables reach a stable limit cycle, then plugging those numerical solutions into expression (12).

Derivation of $R_{0,p}$ and $R_{0,p}^*$ assuming density-dependent transmission

We proceed as before and evaluate whether the density of infected hosts increases or decreases over a single annual cycle. As before, we assume that initially the density of pathogen-infected hosts is small, the population is unvaccinated, and the susceptible population is stably cycling at equilibrium S^*. If we assume that transmission is density-dependent, the equation that determines whether the pathogen-infected density will grow or decline is

$$\frac{dI_p}{dt} = \beta_p\, S^*\, I_p - (d + \gamma_p) I_p. \tag{13}$$

In the density-dependent case, we can write down a solution without linearizing first. Dividing both sides of Eq 13 by I_p, and integrating from time $t = 0$ to $t = T$, the solution can be written as

$$I_p(T) = I_p(0) \exp\left[\int_0^T \beta_p S^* - (d + \gamma_p)\ \mathrm{d}\tau\right]. \tag{14}$$

Once again, the exponent of the exponential function determines whether an initial invasion of the pathogen will increase or decrease over a single annual cycle. The condition for pathogen spread is

$$\beta_p \int_0^T S^* \mathrm{d}\tau - (d + \gamma_p) T > 0 \tag{15}$$

This allows us to conclude that the density dependent form of $R_{0,p}$ is

$$R_{0,p} = \frac{\beta_p \overline{S}}{d + \gamma_p}, \tag{16}$$

where $\overline{S} = \frac{1}{T}\int_0^T S^* \mathrm{d}\tau$ is the average density of susceptible hosts during one annual cycle. In the Mathematica supplementary file, we derive that the average density of susceptibles is related to demographic parameters as $\overline{S} = \frac{b_0 t_b}{dT}$. If the host population is not vaccinated, then $S^* = N^*$, where N^* tracks the total population size throughout the stable limit cycle. Thus, Eq (16) can be written as

$$R_{0,p} = \frac{\beta_p \overline{N}}{d + \gamma_p}, \tag{17}$$

Proceeding as above, we derive that the realized reproduction number satisfies

$$R^*_{0,p} = \frac{\beta_p}{d + \gamma_p}\left(\overline{S} + \overline{S}_v\right) \tag{18}$$

$$= \frac{\beta_p}{d + \gamma_p}\left(\overline{N} - \overline{V}\right) \tag{19}$$

$$= \frac{\beta_p}{d + \gamma_p}\overline{N}(1 - \frac{\overline{V}}{\overline{N}}). \tag{20}$$

Thus, under density-dependent transmission, the fractional reduction in the pathogen's rate of invasion is

$$f_{dens} = \frac{\overline{V}}{\overline{N}} \tag{21}$$

Supplemental figure S1 shows how f_{dens} varies with host lifespan and birthing season. Similar to the case of frequency-dependence, the optimal time for vaccination is at the end of the birthing season.

References

Keeling, M. J. and Rohani, P. (2011), *Modeling infectious diseases in humans and animals.* Princeton University Press
